# Above-canopy versus below-canopy nitrogen addition affects nitrate leaching and mineralization but not greenhouse gas fluxes in a sessile oak stand

**DOI:** 10.1038/s41598-026-36532-z

**Published:** 2026-03-03

**Authors:** Luca Da Ros, Bortolazzi Anna, Panzacchi Pietro, Rodeghiero Mirco, Tognetti Roberto, Mondini Claudio, Fornasier Flavio, Tonon Giustino, Ventura Maurizio

**Affiliations:** 1 Faculty of Agricultural, Environmental and Food Sciences, Free University of Bozen-Bolzano, Piazza Università, 1, 39100 Bolzano, Italy; 2 Department of Agricultural, Environmental, and Food Sciences, University of Molise, Via Francesco De Sanctis, 86100 Campobasso, Italy; 3 Research and Innovation Centre, Fondazione Edmund Mach, Via E. Mach, 1, 38098 San Michele all’Adige, TN Italy; 4 CREA Research Centre for Viticulture and Enology, Via Trieste, 23, 34170 Gorizia, Italy

**Keywords:** N deposition, Canopy nitrogen uptake, Leaf nitrogen uptake, Canopy added nitrogen, Nitrogen saturation process, Element cycles, Biogeochemistry

## Abstract

Increasing nitrogen (N) deposition may alter soil N status and dynamics, as well as the emission of soil greenhouse gases (GHGs). Most of the experimental N manipulations performed so far have neglected the interaction with the canopy, which influences both quantity and quality of the N input into the soil. Here, we assess the effects of N fertilizer application method on N mineralization, and soil GHG fluxes. The experimental site is a sessile oak (*Quercus petraea* L.) stand in Northern Italy and consists of a set of three plots, replicated three times. In each replication, one plot is not fertilized (control plot); one plot receives the fertilization on the forest floor (below-canopy treatment), and one plot receives the fertilization above the canopy (above-canopy treatment). After 5 years of experimental N applications, equal to 20 kg N ha^−1^ y^−1^ distributed equally five times during the vegetative season, net soil N mineralization was assessed with the in-situ soil core incubation method. Soil CO_2_ flux was measured with a portable infra-red gas analyzer, while the soil CH_4_ and N_2_O fluxes were assessed using static closed chambers. No treatment effect was evidenced on soil mineral N content. However, during the last two vegetative seasons, topsoil N leaching increased in the treatment below, and not in the treatment above. On the contrary, N mineralization was lower compared to the control only in the treatment below. These results indicate that the tree canopy can mitigate the effect of N deposition on soil N cycling, which may therefore have been overestimated in previous studies using ground N fertilization. On the other hand, differences in soil GHG fluxes among treatments were not significant, even when the effect of soil temperature and soil moisture was considered. Nevertheless, given the complex relationships between N depositions, soil N dynamics and GHG emissions, long-term investigation is needed to determine whether the presence of the forest canopy, and/or differences in forest type, can mitigate or delay N saturation in the medium to long term.

## Introduction

In recent decades, human activities have roughly doubled global inputs of reactive nitrogen (Nr) to the biosphere^1^, elevating atmospheric N deposition, with hotspots > 50 kg N ha^−1^ y^−1^ in some regions^2^. In Europe, deposition peaked in the 1970s, driving widespread soil acidification and lake eutrophication; since then, anthropogenic N emissions and deposition have declined but remain high in many areas and are even increasing locally^3,4^. These continued N inputs reach ecosystems typically considered N-limited, such as boreal and temperate forests^5^.

Anthropogenic N deposition may have contrasting effects on forest ecosystems^6^. On one hand, increasing N input may enhance the biomass productivity and carbon (C) sequestration^7^, whereas, on the other hand, the extent of these effects is controversial^8^. Carbon accumulation is greater in the wood (60% of N-induced C accumulation) than in the soil (40%)^9^. However, it has been assessed that C accumulation in soils is equal or greater than in plants, due to a suppression of the organic matter decomposition^10^. Despite the uncertainty about which sink, trees or soil, is more affected by N deposition, the consequences of N deposition will influence the C cycle, due to the strong coupling between N and C cycles in forest ecosystems.

An extra Nr input in the N cycle is thought to increase soil N content and affect processes connected with its transformation^11–13^ or lead to nutrient imbalance^14^. These processes (nitrification and mineralization), in turn, influence the soil N pool that is available to plants and the microbial community^13^. The rate of mineralization depends on soil N content^15^ and, therefore, high N deposition may stimulate the rate of ammonification and nitrification^16^. On the other hand, nitrification and denitrification eventually produce nitrous oxide (N_2_O) as by-product, although denitrification may further transform N_2_O into N_2_^17^. Nitrous oxide is an important GHG emitted from soil. Although it is emitted in small amounts, this gas has a very high global warming potential (GWP), 296 times more than CO_2_^18^, also causing the depletion of stratospheric ozone^19^. Soils are estimated to emit globally 6–7 Tg N y^−1^ as N_2_O^20^. In temperate forests, soil N_2_O flux ranges from − 0.5 to 7.3 kg N_2_O-N ha^−1^ y^−1^^21^. The extent of N_2_O flux depends on N availability; therefore, high N deposition may enhance N_2_O flux^22^. Increased N_2_O flux, in combination with N leaching, can be considered an indicator of occurring soil N saturation^23^.

High N deposition may eventually influence the exchange of GHGs between soils and the atmosphere, offsetting the positive effect of N supply on C accumulation and, consequently, on the mitigation potential of forests. The most important GHG emitted by soil is CO_2_, which is the result of the respiration of both microbial communities (heterotrophic) and root systems (autotrophic). Studies have reported either stimulation^24^ or an inhibition^25–27^ in soil CO_2_ flux under increased N deposition. Yet, the effect of N deposition on soil CO_2_ flux can be rate-dependent^28^. Minor changes in soil CO_2_ flux under N deposition may have a significant effect on the global C cycle^29^, since the release of CO_2_ represents a C loss from the soil and variations in soil CO_2_ flux may influence the capacity of soil C sequestration.

Methane (CH_4_) is the second most important GHG, with a GWP 28-times higher than that of CO_2_^18^. Soil is considered a large CH_4_ sink^30^ and forest soils have been estimated to contribute most to global CH_4_ uptake, with an annual absorption of 9.16 Tg CH_4_^31^. Soil CH_4_ flux is, in fact, the result of two processes: production and uptake. Methane production by methanogenetic microbes prevails in poorly drained soils with anaerobic conditions, while CH_4_ uptake occurs in aerobic conditions because of the activity of methanotrophic microbial species^32^. Nitrogen deposition inhibits methanotrophic bacteria in forest soils by altering soil pH and nutrient balances, leading to decreased methane uptake. Therefore, higher quantity of CH_4_ may persist in the atmosphere^33–35^.

Despite the interest in understanding the effects of N deposition on soil GHG fluxes in the context of climate change, uncertainties remain due to different approaches used in the studies performed in this field. First, experiments or observations have been done at different N deposition and/or fertilization rates (from lower than 30 to higher than 60 kg N ha^−1^ y^−1^)^36^. Furthermore, most manipulative studies have simulated higher N deposition by applying fertilizers directly on the forest floor, with only few of them integrated canopy N application^13,37^. However, ground N application neglects the potential interactions of N deposition with tree canopy, which has been shown to play an active role in the N cycle, by retaining atmospheric N and partly preventing its arrival to the soil^38–41^. Retained N can be absorbed by plants and used for satisfying plant N demands^42^. In addition to the canopy pathway, microbial activities, such as nitrification^43^, ammonia oxidation^44^, and N fixation^45^ occur within the canopy, leading to a change in the chemical form of the deposited N. Excluding canopy interactions with N deposition in experimental designs may result in biased outcomes^36^. The canopy plays a crucial role in intercepting and processing atmospheric N, potentially buffering soil N input and influencing GHG emissions. Neglecting this component risks an incomplete understanding of N cycling and its impact on soil GHG dynamics in forest ecosystems. For this reason, it is very important to apply N fertilizer above the canopy if we want to assess the effect of this element on forest ecosystems.

To understand the effects of extra N input on soil N mineralization and soil GHG fluxes in a temperate forest, we established a field manipulation experiment in summer 2014, comparing N fertilization above and below the forest canopy^39^ as very few authors have done so far^13,38^, and for the first time in a sessile oak (*Quercus petraea* L.) forest. Here, we aim to determine the quantitative effect of canopy-interception of increased N deposition on soil N mineralization, leaching, and soil GHG fluxes. We propose that the position of N application (above vs. below the canopy) significantly influences its impact on forest-floor biogeochemistry. When fertilizer is spread above the canopy, a substantial fraction is intercepted by foliage, whereas below-canopy applications deliver nearly the entire dose directly to the soil. Consequently, our hypotheses are: (1) below-canopy application will raise soil N availability much more than above-canopy application, potentially leading to greater N leaching; (2) the different surge in available N will stimulate microbial respiration and nitrification, resulting in treatment-specific increases in CO_2_ and N_2_O; (3) at the same time, the rise in soil NH_4_^+^ from below-canopy application will likely suppress CH_4_ oxidation through substrate competition, diminishing the soil’s CH_4_ sink strength—a response expected to be weak or absent under above-canopy application because of the limited delivery of NH_4_^+^ to the soil.

In short, canopy interception acts as a buffer that moderates N-driven effects; bypassing this buffer through below-canopy fertilization amplifies those effects.

## Materials and methods

### Experimental site

The study was conducted in the experimental site of Monticolo, in Northern Italy (46° 25′ 35″ N; 11° 17′ 55″ E) at 530 m a.s.l. In Monticolo, the annual average temperature is 11.4 °C, the average annual precipitation is 800 mm, and the atmospheric N bulk deposition was 5.1 kg N ha^−1^ y^−1^ through the experimental period^14^. The soil is acid brown (Cambisol, WRB_2015_, IUSS Working Group, 2015), from porphyritic quartz rock^46^. The forest is dominated by sessile oak (*Quercus petraea* L.) (95%), whereas other sporadic species are Scots pine (*Pinus sylvestris* L.), lime (*Tilia cordata* Mill.), chestnut (*Castanea sativa* Mill.), European hop-hornbeam (*Ostrya carpinifolia* Scop.), and silver birch (*Betula pendula* Roth). A complete description of the stand characteristics is available in^47^. The stand grows on a shallow Cambisol with a pH value of 5.5 and a loam texture (45% sand, 46% silt, 9% clay), over a quartz porphyritic bedrock. Foliar N concentration observed in control plots in a previous study (1.98 ± 0.05%) suggests that this oak forest is N-limited, consistent with findings indicating N limitation for sessile oak^39,48^.

### Experimental treatments

The experimental design consists of nine plots of a 12-m radius, three for each treatment. The plots are randomly distributed in a 200-m-long area to minimize the variation in forest conditions. In addition, a buffer distance of at least 10 m between plots avoids contamination during the application of N fertilizer. The plots were chosen in relatively homogeneous areas, minimizing the presence of pits or mounds or other sources of variations in soil morphology.

Treatments are unfertilized plots (control), fertilization above the canopy (“above” hereafter), and fertilization on the forest floor (“below” hereafter). Fertilization above is achieved through a sprinkler (Rain Bird SNC, Aix-en-Provence, France) assembled on the top of a telescopic pole (model Maxi Primo, Fireco S.R.L., Gussago, Brescia, Italy). The required pressure for the water solution to reach the sprinkler is provided by a pump driven by a gasoline powered engine (mod. 46A, Officine Carpi S.R.L., Poviglio, Reggio Emilia, Italy). Sprinklers reach 15–18 m height, according to tree height, and spread the fertilizer solution for a 12-m radius. Fertilization below is made manually, with a water hose and a spray nozzle, paying attention to distributing the fertilizer solution uniformly on the forest floor. Care was taken to apply treatments in no wind and no rain conditions when the weather forecast did not predict any rain event for at least the following 24 h (to prevent leaf wash-off). Fertilization has been ongoing at the site since 2015; with applications from May to September at monthly intervals (i.e., five per growing season). The fertilization compound is a solution of NH_4_NO_3_ (4.3 g N/L) and the annual fertilization rate is 20 kg N ha^−1^ y^−1^ (i.e., 4 kg N ha^−1^ per single application) in both above and below treatments. This is equivalent to almost four times the background bulk N deposition in the area^49^ and about 3.3 times of the global average rate of total N deposition to forests (6 kg N ha^−1^ y^−1^)^50^. The experimental design, procedure, and timing are described in detail in previous publications^39,47^, additional schematic drawings and methodological details can be found there. The amount of water provided annually with the solution is equivalent to precipitation of 0.46 mm, hence, negligible when compared to the average annual precipitation. Consequently, this quantity was not administered to the control plots.

**Fig. 1:**
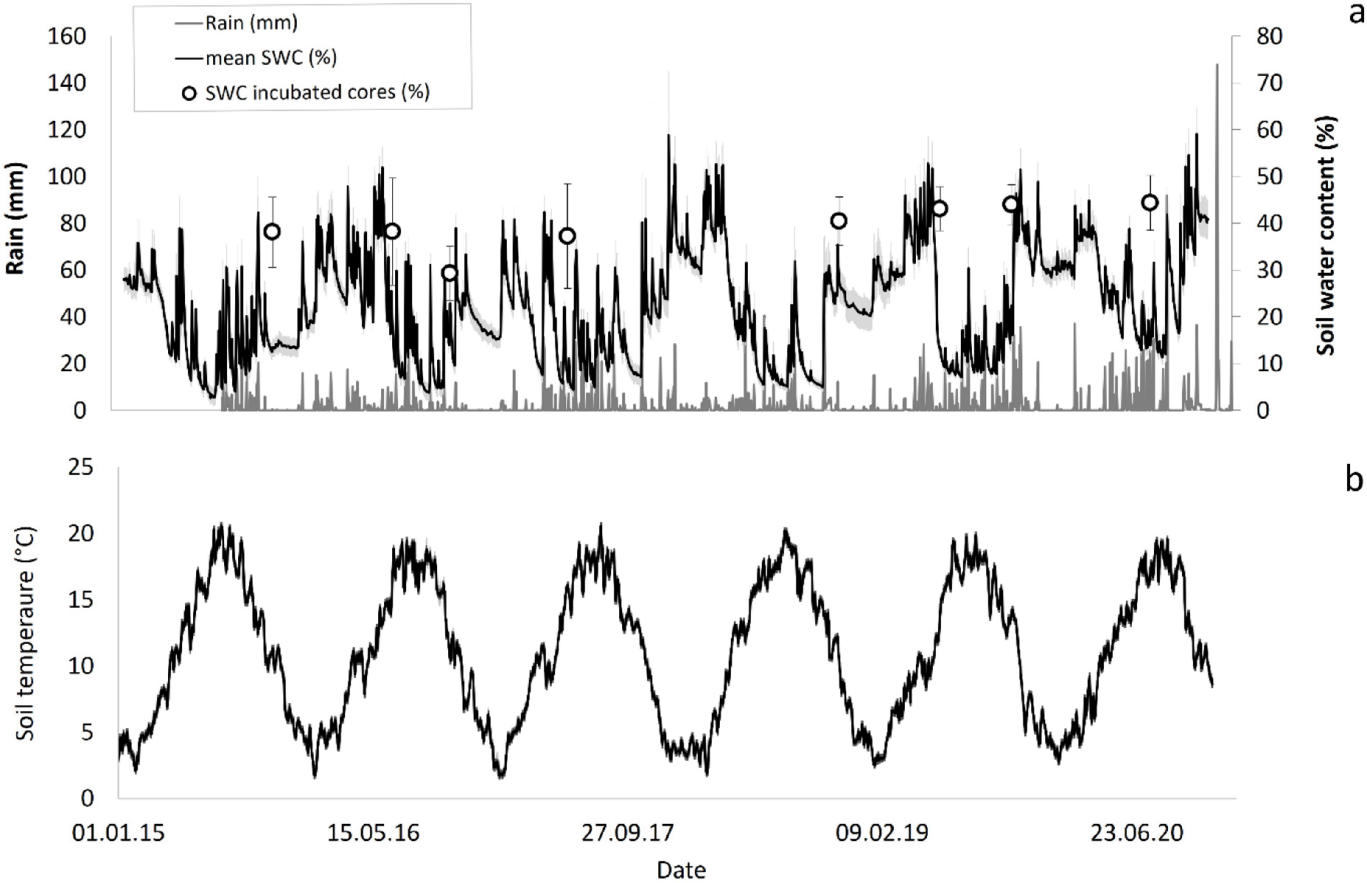
Rain events and mean soil water content (SWC, **a**) and soil temperature (**b**) in the experimental site, during the experimental period. Empty circles indicate the SWC of the incubated samples; the error bars indicate the standard error of the mean.

In each plot, soil temperature at 10 cm depth and soil water content (SWC) at 5 cm depth are measured close to the plot center with a HOBO 12-bit Temperature Smart Sensor (Onset Computer, Bourne, MA, USA) and an ECHO-20 probe (Decagon Devices, Pullman, WA, USA), respectively, connected to a HOBO Micro Station data logger (Onset Computer, Bourne, MA, USA). The soil moisture probe was specifically calibrated for the soil of the experimental site against gravimetric water content. Measurements are performed every 3 min and averaged every 30 min (Fig. 1).

### Net N mineralization and leaching

**Fig. 2:**
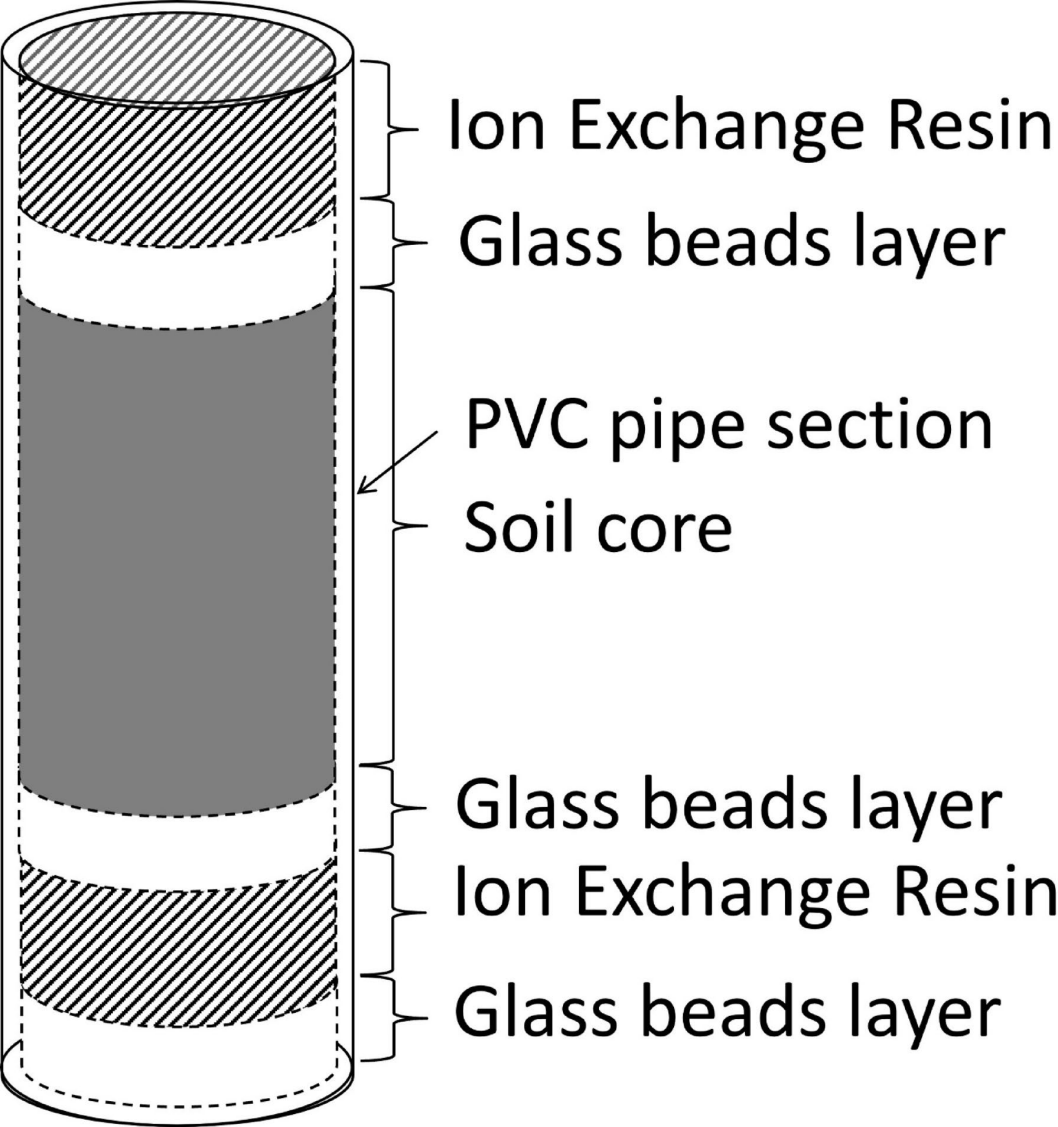
Representation of in-situ soil incubation system.

Net N mineralization and leaching were assessed using the in-situ soil core incubation method^51^. In each plot, three soil cores were collected by hammering a PVC pipe section (5 cm diameter, 5 cm height) in the forest floor, after removal of the litter. The cores, stored in plastic bags, were brought to the laboratory and kept in the fridge until analysis to determine the initial NO_3_-N and NH_4_-N content. Other three cores were collected (each one close to the first one), inserted in a PVC pipe section, and interposed between two 10-g ion-exchange resin layers (DOWEX™ MARATHON™ MR-3), placed on the top and at the bottom of the soil core (Fig. 2). The top resin layer prevented mineral N from entering the core, while the bottom resin layer collected the N ions leaching from the soil core. A layer of glass beads (2 mm diameter) was placed between each resin layer and the soil core, to avoid contamination of resin with soils. The soil, the ion-exchange resin layers, and the glass bead layers were separated by a 125-µm mesh nylon net (Scubla s.n.c., Remanzacco, UD, Italy). The second core was, then, inserted in the soil for one month, to keep it at the same temperature as that of natural conditions. The incubation process was repeated seven times throughout the study, with each incubation lasting one month. The incubation periods concluded on the following dates: 13 November 2015; 5 July 2016; 26 October 2016; 13 July 2017; 12 June 2019; 30 October 2019; and 28 July 2020. Soil N mineralization was measured in autumn and in spring and early summer, as in these seasons we expected to have high N mineralization because of favorable environmental conditions (sufficiently high soil water availability and soil temperature). We also wanted to minimize the influence of residual N from fertilization, as we wanted to assess the effect of N deposition due to a long-lasting change in N dynamics in soil and not on the temporary effect of N from treatments. Therefore, the soil cores were incubated at the beginning of the fertilization treatments and after about one month from the last treatment. At this time, we expected a minimal presence of residual N in soil from fertilization, even though this effect could not be completely excluded.

In the lab, the initial and incubated soil samples were weighed and sieved at 2 mm to remove stones and coarse organic matter fragments, which were weighed as well. A subsample was collected from the sieved soil and oven-dried at 105 °C for 24 h, to determine SWC. The dry weight of the soil in each core was determined on the base of the fresh weight of the core and the SWC, after subtracting the weight of the stones and coarse organic matter fragments. Another subsample (about 5 g) of fresh sieved soil was mixed with 50 ml of a 2M KCl solution. The solution was shaken for two hours and filtered with Whatman 42 filter paper. Resins from the bottom core layers were extracted with 100 ml of 2M KCl solution following the same procedure used for soils. The NO_3_-N and NH_4_-N content of the extraction solutions (of both soil and resin) was determined with a colorimetric analyzer (AxFlow AA3, Bran + Luebbe, Norderstedt, Germany), using salicylate and dichloro-isocyanuric acid method for ammonium (ISO 11732:2005) and sulfanilamide–NEDD [N-(1-Naphthyl) ethylenediamine] reaction for nitrate (ISO 13395:2006). Topsoil leaching of NO_3_-N and NH_4_-N was determined from the NO_3_-N and NH_4_-N content in the bottom resin layer. The NO_3_-N and NH_4_-N content of the extraction solutions were used to calculate the soil NO_3_-N and NH_4_-N content (dry weight). Values in mg N g^−1^ soil were used to calculate the net variation of nitrate-N (ΔNO_3_-N) and ammonium-N (ΔNH_4_-N), and the total N mineralization rate, using the following equations^12^:

1 $$\Delta {\mathrm{NO}}_{3} {\mathrm{-N}} = \left( {{\mathrm{NO}}_{3} {\mathrm{-N}}_{{{\mathrm{initial}}}} - {\mathrm{NO}}_{3} {\mathrm{-N}}_{{{\mathrm{final}}}} } \right) + {\mathrm{NO}}_{3} {\mathrm{-N}}_{{{\mathrm{resin}}}}$$

2 $$\Delta {\mathrm{NH}}_{4} {\mathrm{-N}} = {\mathrm{NH}}_{4} {\mathrm{-N}}_{{{\mathrm{resin}}}} + \left( {{\mathrm{NH}}_{4} {\mathrm{-N}}_{{{\mathrm{resin}}}} - {\mathrm{NH}}_{4} {\mathrm{-N}}_{{{\mathrm{final}}}} } \right)$$

3 $${\mathrm{Nmineralization}} = \Delta {\mathrm{NO}}_{3} {\mathrm{-N}} + \Delta {\mathrm{NH}}_{4} {\mathrm{-N}}$$

where NO_3_-N_initial_ and NH_4_-N_initial_ are the initial NO_3_-N and NH_4_-N content in the soil, respectively; NO_3_-N_final_ and NH_4_-N_final_ are the soil NO_3_-N and NH_4_-N content after the incubation; NO_3_-N_resin_ and NH_4_-N_resin_ are the NO_3_-N and NH_4_-N content in the bottom resin layer after incubation.

### Soil CO_2_ flux

For the measurement of soil CO_2_ flux, three collars made from PVC pipe (10 cm height, 8 cm diameter) were inserted 5 cm into the soil in each plot, for a total of 27 sampling points. To avoid the influence of the mechanical disturbance of soil due to collar insertion, these PVC pipes were inserted one week prior to the first measurement. Soil CO_2_ flux (g CO_2_ m^−2^ h^−1^) was measured with a portable infra-red gas analyzer (EGM 4, PP Systems, UK) connected with a closed dynamic chamber (SRC 1, PP Systems, UK). Measurement time was set to 80 s. The collars could have potentially affected soil moisture due to the shading on soil topsoil of the portion about 5 cm above the soil topsoil. However, this effect could be significant on bare soil but is likely minimal in temperate vegetated area^52^. Therefore, we think that in Monticolo forest collars did not significantly affect the environmental drivers of GHG emission from soil.

During soil respiration measurements, soil temperature (°C) at 10 cm depth and SWC (m^3^ m^−3^) at 5 cm depth were measured close to the collar with a temperature probe (STP 1, PP Systems, UK) and a soil moisture probe (Type ML2x, Delta-T devices Ltd., Cambridge UK), respectively. The soil moisture probe was specifically calibrated for the soil of the experimental site against gravimetric water content. The measurements were performed on average every 6 weeks from March 2018 to August 2020, between 11.00 and 14.00.

### Soil CH_4_ and N_2_O fluxes

Soil CH_4_ and N_2_O fluxes were measured with the method of the closed static chambers^53^. Each chamber was made from a PVC collar (diameter 25 cm, height 8 cm), closed with a removable top and neoprene seal, to make it airtight. On the top, a circular hole (12 mm) was drilled, where a pierceable septum was installed. Preliminary laboratory tests were performed to assess the gas-tightness of chamber sealing. One of the chambers was put in a plastic tray with a 1-cm water layer, to avoid gas leaking from the bottom. Then, 50 ml of pure acetylene gas was injected into the chamber through the septum after the removal of the same amount of air from the chamber to avoid an increase in pressure. Acetylene concentration in the chambers was measured at 5- to 10-min intervals until 30 min from injection with a gas-chromatograph (GC). As the acetylene concentration in the chamber was constant, no considerable gas leak was detected from the chamber during the test.

In May 2019, three permanent collars were installed in each plot, for a total of 27 collars. The collars were inserted 3 cm into the soil, leaving 5 cm above the soil. On each measurement day (31 May 2019, 7 June 2019, 26 June 2019, 26 August 2019, 27 September 2019, 30 October 2019, 21 January 2020, 24 March 2020, 29 April 2020, 22 June 2020, 29 July 2020, 30 August 2020, and 26 November 2020), the removable lids were placed on the collars, to create closed chambers. Immediately after chamber closure, a 10-ml air sample was collected with a syringe from each chamber and transferred to a 12-ml Exetainer® glass vial (Labco Ltd., UK), previously flushed with N_2_. The other three air samples were collected from the chambers at 5, 10, and 15 min from closure. The exact time of collection was recorded.

Collected air samples were analyzed at the laboratory of CREA-VE (Council for Agricultural Research and Economics, Research Centre for Viticulture and Enology, branch of Gorizia, Italy), with a GC equipped with an electron capture detector (ECD) to valuate N_2_O concentration and with a flame ionization detector (FID) to determine CH_4_ concentration. Calibration gas standards were prepared by injecting gas mixtures from certified gas cylinders in the flushed exetainers. Standards were analyzed by means of the same procedure used for chamber air samples and for instrument calibration. Fluxes of CH_4_ and N_2_O from soil were calculated from the slope of the regression line between gas concentration and its change over time in the chambers, determined in the collected samples at the different sampling times^53^.

### Data elaboration and statistical analysis

We analyzed the effect of fertilization treatments and sampling time on soil nitrogen transformation processes using a repeated measures linear mixed-effects modelling (LMM) framework, implemented via the *lme* function from the *nlme* package^54^. All statistical analyses were performed in R (version 4.3.3; R Core Team, 2024), following best practices for ecological data analysis^55^. Measurements obtained from different points within each plot were averaged to provide a single value per plot, which was considered the experimental unit. Treatment and time, as well as their interaction, were included as fixed effects, while plot was incorporated as a random effect to account for within-plot correlations over time^56^.

Model assumptions of normality and homogeneity of variance were assessed through visual inspection of residuals plotted against treatment, date, and plot factors^55^. When violations of homoscedasticity were detected, we applied an appropriate variance structure using the *varIdent* function to allow for heterogeneous variances among treatment and time combinations, which improved model fit^55^.

Once the final model was validated, the *anova* function was used to evaluate the significance of the main effects and their interaction^54^. In cases where a significant treatment-by-time interaction was found, post-hoc pairwise comparisons were performed using Tukey’s test with the *emmeans* package^57^, applying multiple comparison adjustments^56^.

Analysis of soil GHG fluxes was done using linear models, in which soil temperature, SWC, and fertilization treatment (above, below, and the unfertilized control) were used as independent variables. Additionally, also the influence of soil litter biomass and soil C stock on soil CO_2_ flux was analyzed after inclusion in the model. Measured fluxes of GHG, soil temperature, and water content were averaged at the plot level before analysis. For each treatment, the relation between soil CO_2_ flux and soil temperature was fitted using a simple exponential model:

4 $${\mathrm{SR}} = {\mathrm{R}}_{0} e^{{{\mathrm{bT}}}}$$

where SR is the soil CO_2_ flux, T is soil temperature (°C) measured at 10 cm depth near the collars where SR was measured, R_0_ is the basal respiration rate and *b* is a model coefficient. R_0_ and *b* were estimated by a non-linear regression procedure, using the *minpack-lm* package (v1.2-1). This model is most used to describe the dependence of soil CO_2_ flux on soil temperature^58^.

To better isolate the effect of soil temperature on soil CO_2_ flux, data corresponding to limiting SWC values (< 0.07 m^3^ m^−3^) were excluded from the analysis. This humidity threshold value was determined visually, after plotting soil CO_2_ flux against SWC. The apparent sensitivity of soil CO_2_ flux to soil temperature was, therefore, determined for each treatment calculating the Q_10_ value (= e^10b^)^59^. Model fit statistics and regression parameters (R_0_ and b) for Eq. (4) are reported in Table 1.

**Table 1:** R^2^, *p* value and parameters (R_0_ and b) for each treatment of the Eq. (4) relating soil CO_2_ flux to the soil temperature.

Treatment	*R* ^2^	*p* value	Regression parameters	
			*R*_0_	*b*
Above	0.55	< 0.01	0.126	0.121
Below	0.65	< 0.01	0.128	0.122
Control	0.55	< 0.01	0.125	0.121

Regression models obtained for the different treatments were linearized by logarithmic transformation and then compared to test for the presence of statistically significant differences in the slope and intercept of the regression lines using the analysis of covariance (ANCOVA).

A second approach was used to assess the response of soil CO_2_ flux to both soil temperature and humidity, by fitting soil CO_2_ flux data from each plot with the following non-linear model^60^:

5 $${\mathrm{SR}} = {\mathrm{a}}\,{\mathrm{T}}^{{\mathrm{b}}} \,\uptheta ^{{\mathrm{c}}} ,$$

where SR is the soil CO_2_ flux, T is soil temperature (°C), θ is the soil water content (m^3^ m^−3^), and *a*, *b* and *c* are regression coefficients calculated for each experimental plot. The model coefficients were calculated in R with the *minpack-lm* package (CRAN), and the significance of the model for each plot was assessed with the summary function of the same package. The mean values of the model parameters derived for the different treatments were compared using the ANOVA, to evaluate whether the response of soil CO_2_ flux to both soil temperature and soil moisture was statistically different according to the fertilization treatment.

The obtained nonlinear models (Eq. 5) were also used to calculate the soil CO_2_ flux from each plot daily, based on the daily average values of soil temperature and SWC, which were measured continuously in each plot. The cumulative annual soil CO_2_ flux was calculated as the sum of daily values for the period from 1 March 2018 to 28 February 2019 and from 1 March 2019 to 29 February 2020.

Relationships of CH_4_ and N_2_O fluxes with soil temperature and soil moisture were analyzed using a linear regression analysis. Considering the time needed to sample chamber air to calculate methane and N_2_O fluxes (about 15 min), the fluxes of these gases were related to soil temperature and water content measured continuously in the center of the plot.

The presence of statistically significant differences between the slopes and intercepts of the regression lines obtained for the different treatments was checked using ANCOVA. Furthermore, the annual flux for each gas and for each plot was calculated by interpolation of the measured fluxes for the period from 26 June 2019 to 25 June 2020. Average values of the annual fluxes were compared with the ANOVA, to highlight the presence of statistically significant differences.

## Results

### Soil N mineralization and leaching

**Fig. 3:**
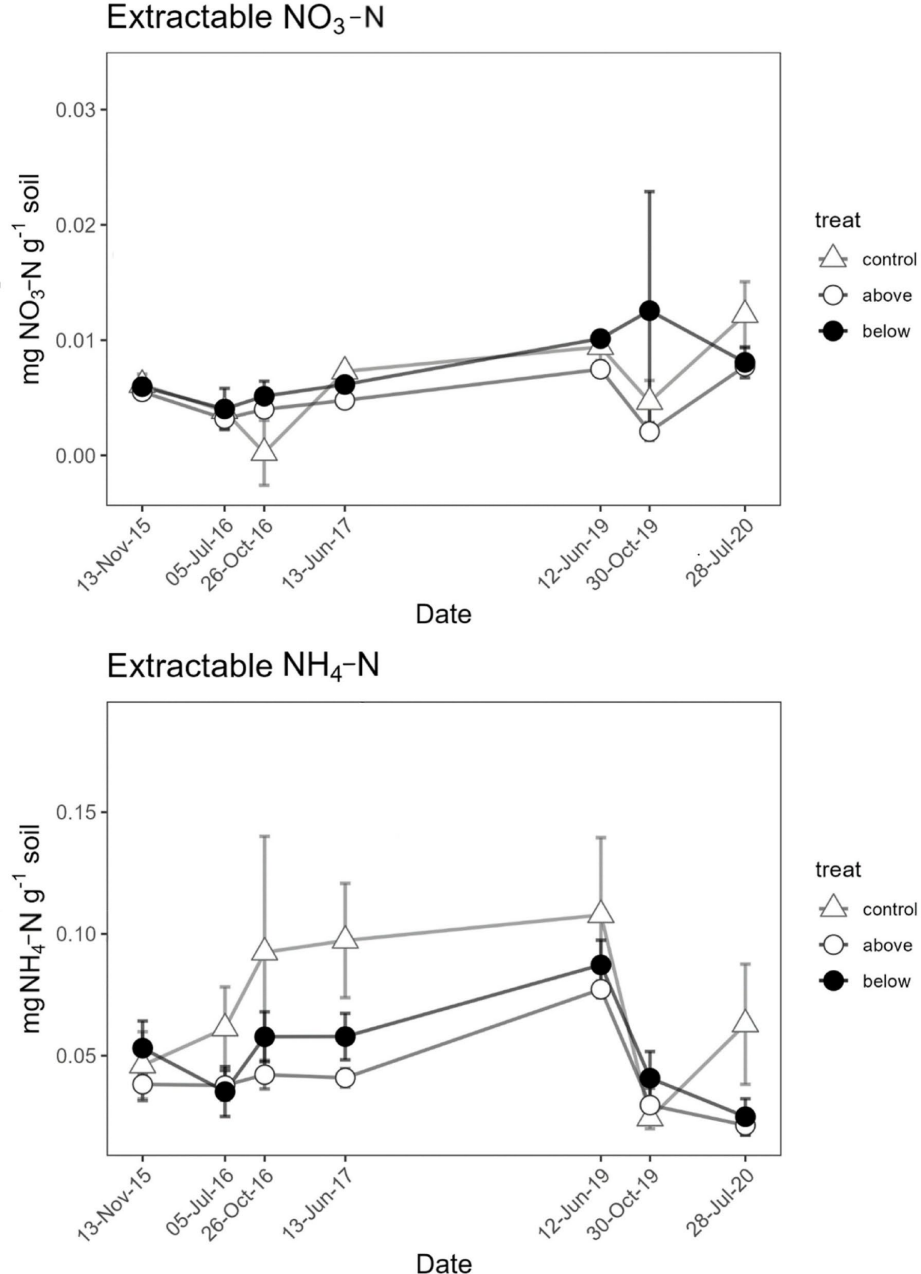
Soil extractable NO_3_-N (**a**) and NH_4_-N (**b**) in the soil before incubation. No significant differences were evidenced among treatments. Data points represent average values of three replicates and vertical bars show the standard error (n = 3).

Extractable NO_3_-N (nitrate) content ranged between 0.002 and 0.013 mg g^−1^ soil (Fig. 3a). Despite a significant overall treatment effect, and the absence of significant interaction between treatment and time, pairwise comparisons did not evidence significant differences between treatment levels (Fig. 3a). Extractable NH_4_-N (ammonium) content ranged between 0.002 and 0.013 mg NH_4_-N g^−1^ soil (Fig. 3b), being higher in spring 2019 than in other periods. Significant interaction between time and treatment was found, but post hoc tests revealed no difference at any given time.

**Fig. 4:**
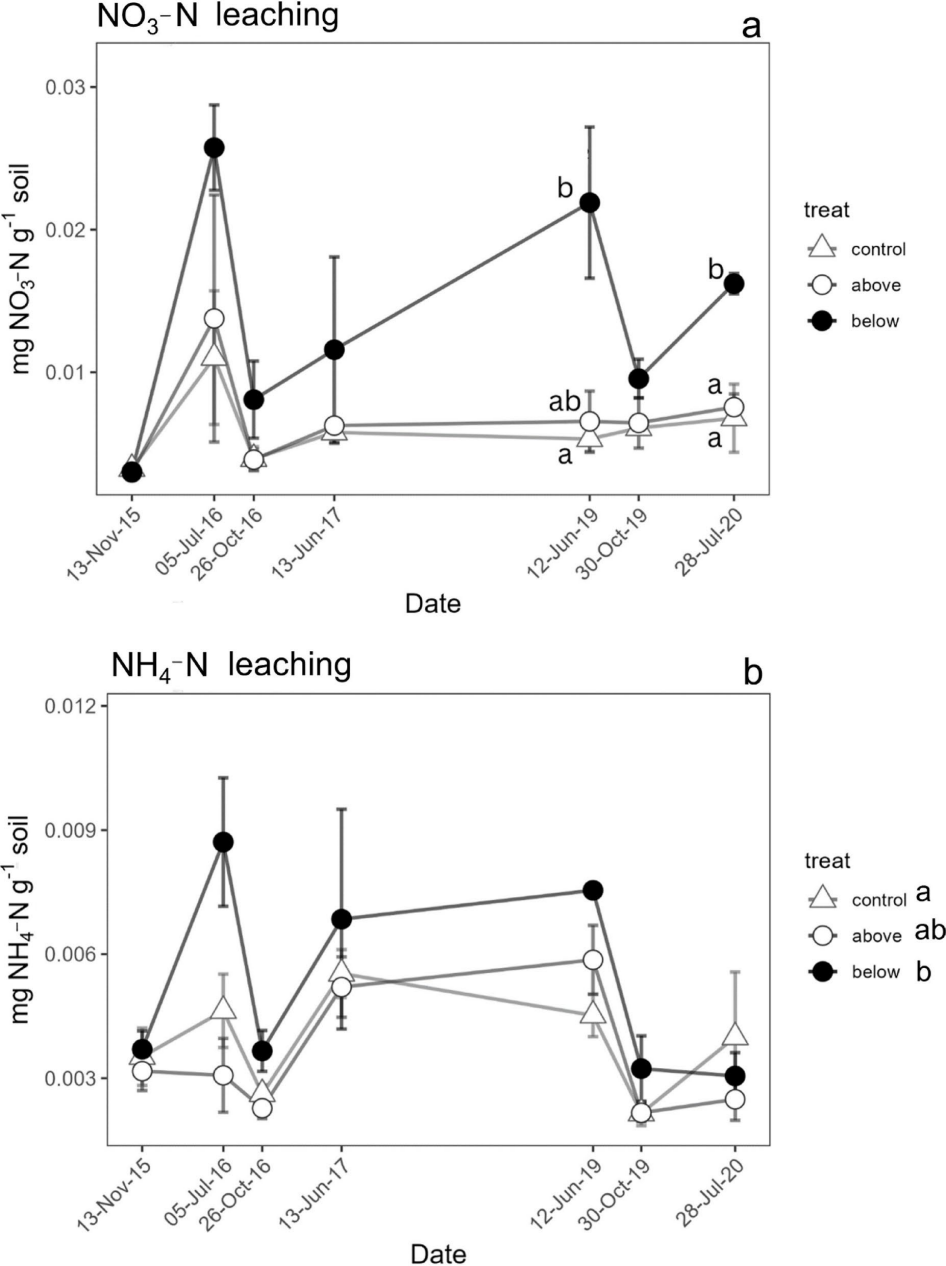
Leaching from topsoil of NO_3_-N (**a**) and NH_4_-N (**b**) during the incubation period. Different letters in the graph indicate significant difference among treatments within single sampling periods (Tukey test, p < 0.05). Different letters in the Figure legend evidence overall significant differences among the treatment levels (in case no interaction was found between treatment and time). Data points represent the average of three replicates, and vertical bars show the standard error (n = 3).

**Fig. 5:**
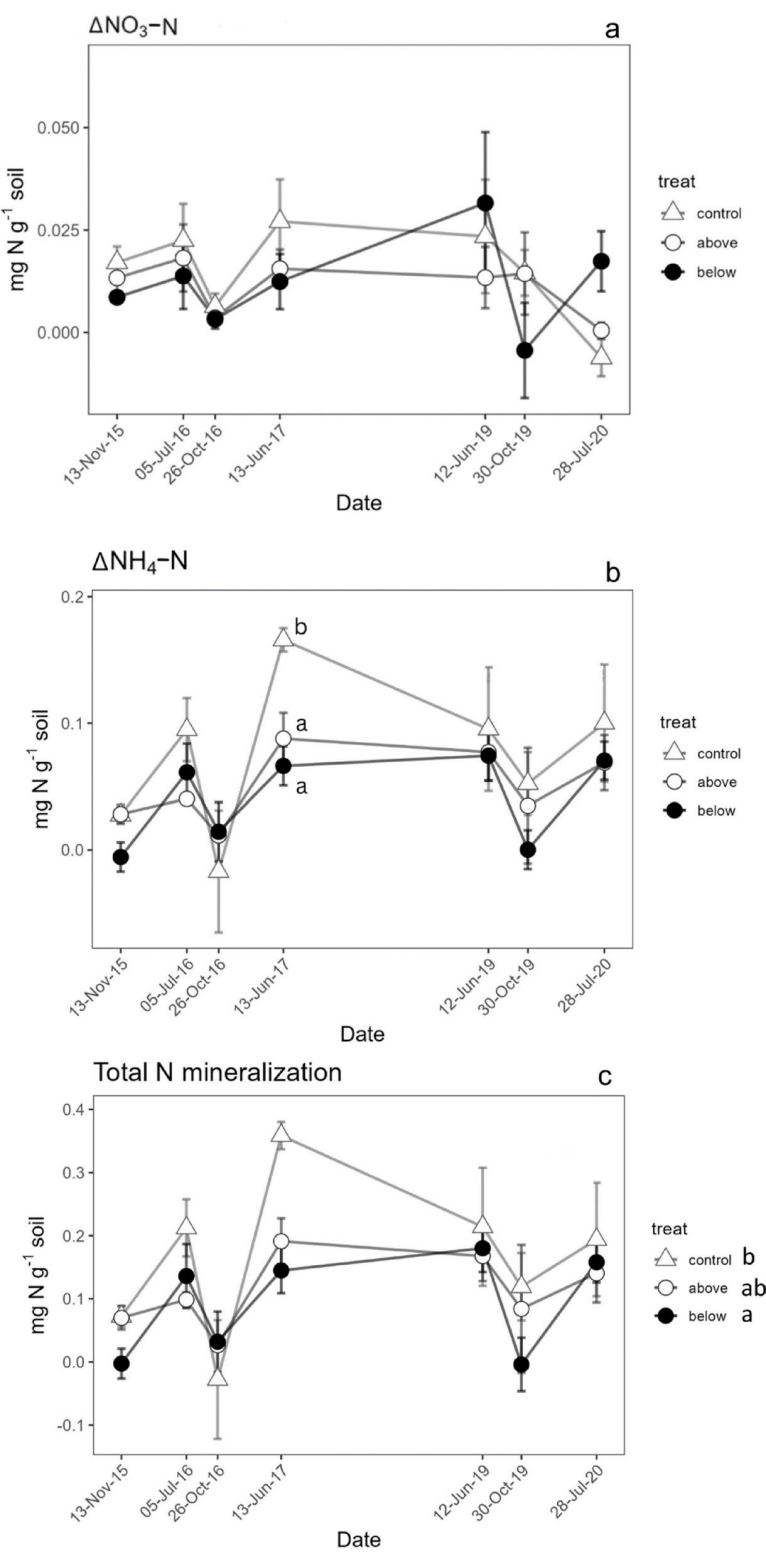
ΔNO_3_-N (**a**), ΔNH_4_-N (**b**), and total N mineralization (**c**) in the different treatments. Δ values were calculated according to Eqs. (1) and (2) to represent net changes relative to initial concentrations. Different letters in the graph indicate significant difference among treatments within single sampling periods (Tukey test, p < 0.05). Different letters in the Figure legend evidence overall significant differences among the treatment levels (no interaction between treatment and time). Data points are average values of three replicates; vertical bars represent the standard error (n = 3).

Topsoil leaching of NO_3_-N was higher in treatment below than in the control in spring 2019 and summer 2020, while the treatment above was not significantly different from the control (Fig. 4a). A significant treatment effect was found for topsoil leaching of NH_4_-N, with the treatment below resulting higher compared to the above treatment, while the control group showed intermediate values (Fig. 4b). The interaction between treatment and date was not significant, indicating the treatment effects were consistent across dates (Fig. 4b). The net variation of ΔNO_3_-N showed no significant differences among treatments during any of the measurement periods (Fig. 5a). In contrast, the net variation of NH_4_-N exhibited a significant difference among treatments only in summer 2017, when the treatment below had higher values compared to both the treatment above and the control (Fig. 5b). Total N mineralization did not show significant interaction between time and treatment (Fig. 5c) and was lower in the treatment below, while the treatment above was not significantly different from the control (Fig. 5c).

### Soil CO_2_ flux

**Fig. 6:**
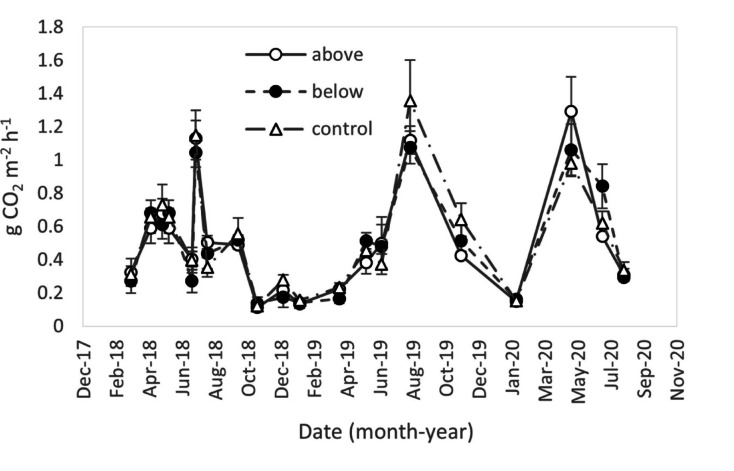
Seasonal trend of CO_2_. Values of soil CO_2_ flux are the average per treatment; bars represent the standard error (n = 3).

**Fig. 7:**
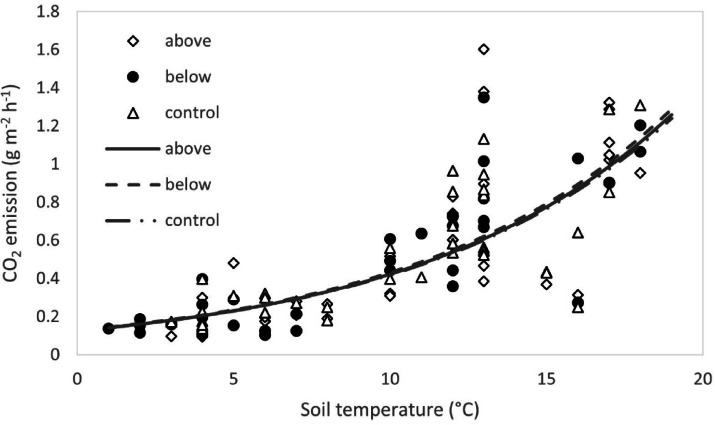
Measured soil CO_2_ flux in relationship with measured soil temperature. Lines represent the exponential model (Eq. 4) for each treatment.

No significant differences were detected between soil CO_2_ flux measured in the different treatments, in any of the measurement days (Fig. 6). Soil CO_2_ flux showed a seasonal variation, with the highest values in summer, when the soil temperature was at its maximum. When SWC was not limiting soil respiration, the exponential model (Eq. 4) fitted soil CO_2_ flux data well (R^2^ = 0.63, *p* < 0.001) (Fig. 7). However, the ANCOVA showed that experimental treatments influenced neither the slope nor the basal respiration R_0_ of the regression with temperature (Fig. 7). Therefore, the N fertilization applied either above or below the canopy did not significantly affect the sensitivity of soil CO_2_ flux to temperature. The Q_10_ values for soil CO_2_ flux were 3.36, 3.37, and 3.35 for above, below, and control treatments, respectively.

Non-linear regression analysis (Eq. 5) showed that both soil temperature and SWC were statistically significant drivers of soil CO_2_ flux for all the experimental plots. The models predicting soil CO2 flux in relation to the environmental variables showed a low RMSE (Table 2). However, the amount of variability explained by the predictors was between 35 and 44% only (Table 2). No significant differences were detected between the model parameters for the different treatments.

**Table 2:** Parameter estimation, RMSE, and R^2^ of the models relating soil CO_2_ flux to soil temperature and soil water content (SWC). Values are the average of three replicates (plots) per treatment ± standard error.

Treatment	RMSE (g CO_2_ m^−2^ y^−1^)	*R* ^2^	Regression parameters		
			*a* (g CO_2_ m^−2^ y^−1^)	*b*	*c*
Above	0.29 ± 0.05	0.44 ± 0.06	0.08 ± 0.03	1.5 ± 0.1	0.88 ± 0.06
Below	0.26 ± 0.03	0.44 ± 0.04	0.11 ± 0.03	1.12 ± 0.08	0.68 ± 0.03
Control	0.33 ± 0.09	0.35 ± 0.05	0.09 ± 0.02	1.20 ± 0.09	0.7 ± 0.1

**Fig. 8:**
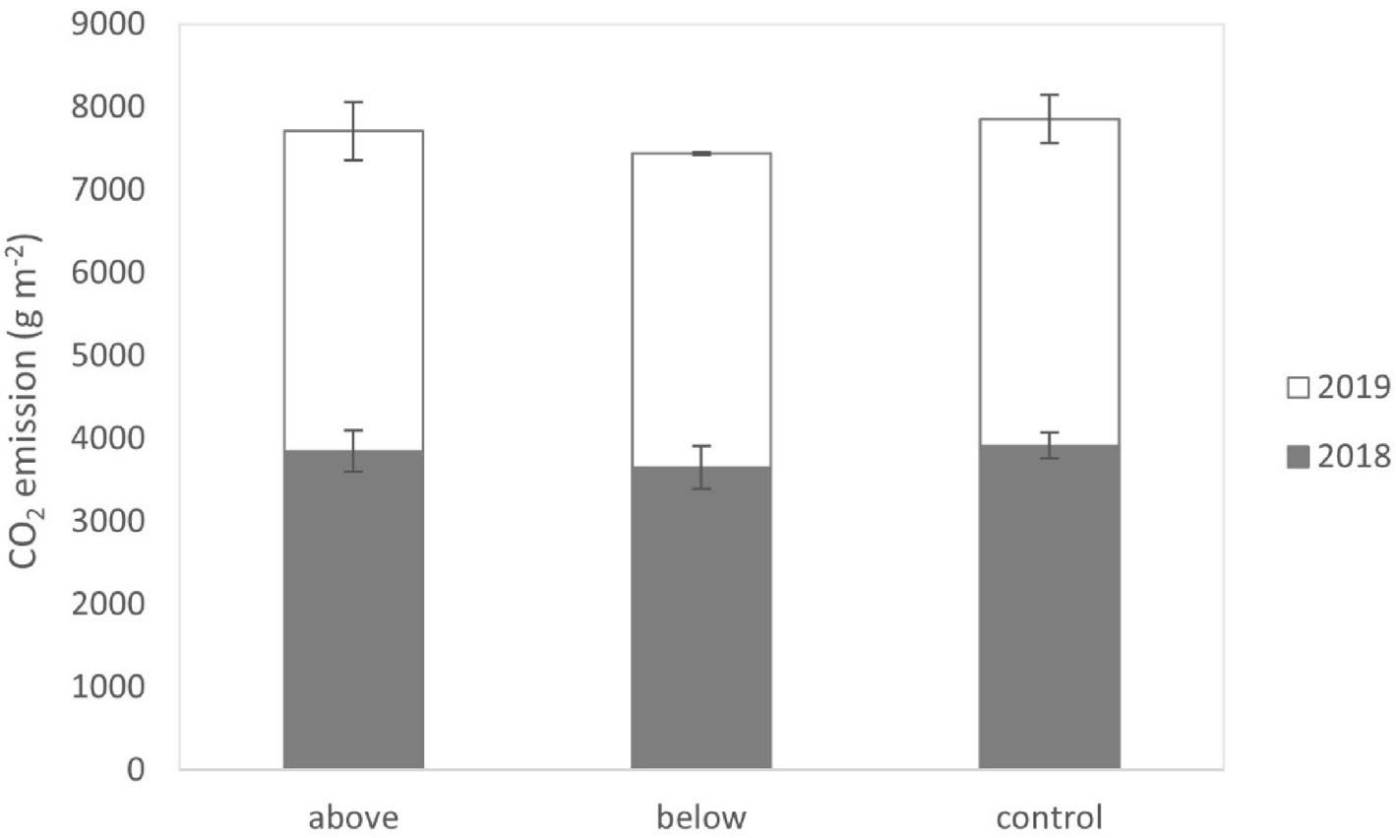
Cumulative annual soil CO_2_ flux for 2018, 2019 (reference period March 1–February 28), and in the two-year monitoring period. Error bars indicate the standard error (n = 3).

The cumulative annual soil CO_2_ flux was not affected by treatments, as well as the total soil CO_2_ flux calculated over the two years of monitoring (Fig. 8). On average, the total soil CO_2_ flux was 3805 g CO_2_ m^−2^ y^−1^ in 2018 and 3862 g CO_2_ m^−2^ y^−1^ in 2019.

### Soil CH_4_ uptake

**Fig. 9:**
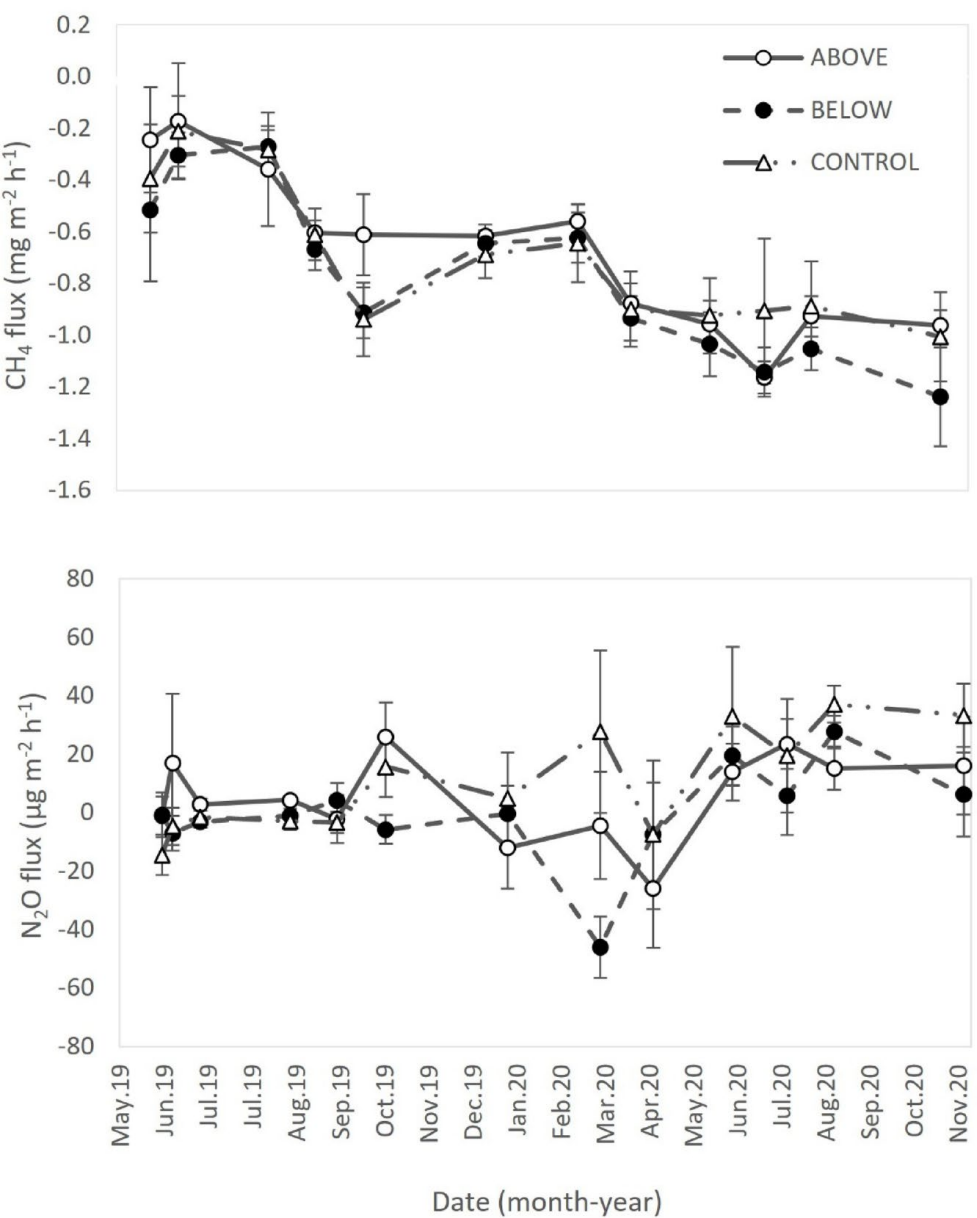
Time trends of soil CH_4_ flux (**a**), and N_2_O emission (**b**). Positive values indicate net soil GHG fluxes from the soil to the atmosphere, while negative values indicate net fluxes from the atmosphere to the soil. Data points represent the average of three replicates per treatment. Error bars represent the standard error (n = 3).

**Fig. 10:**
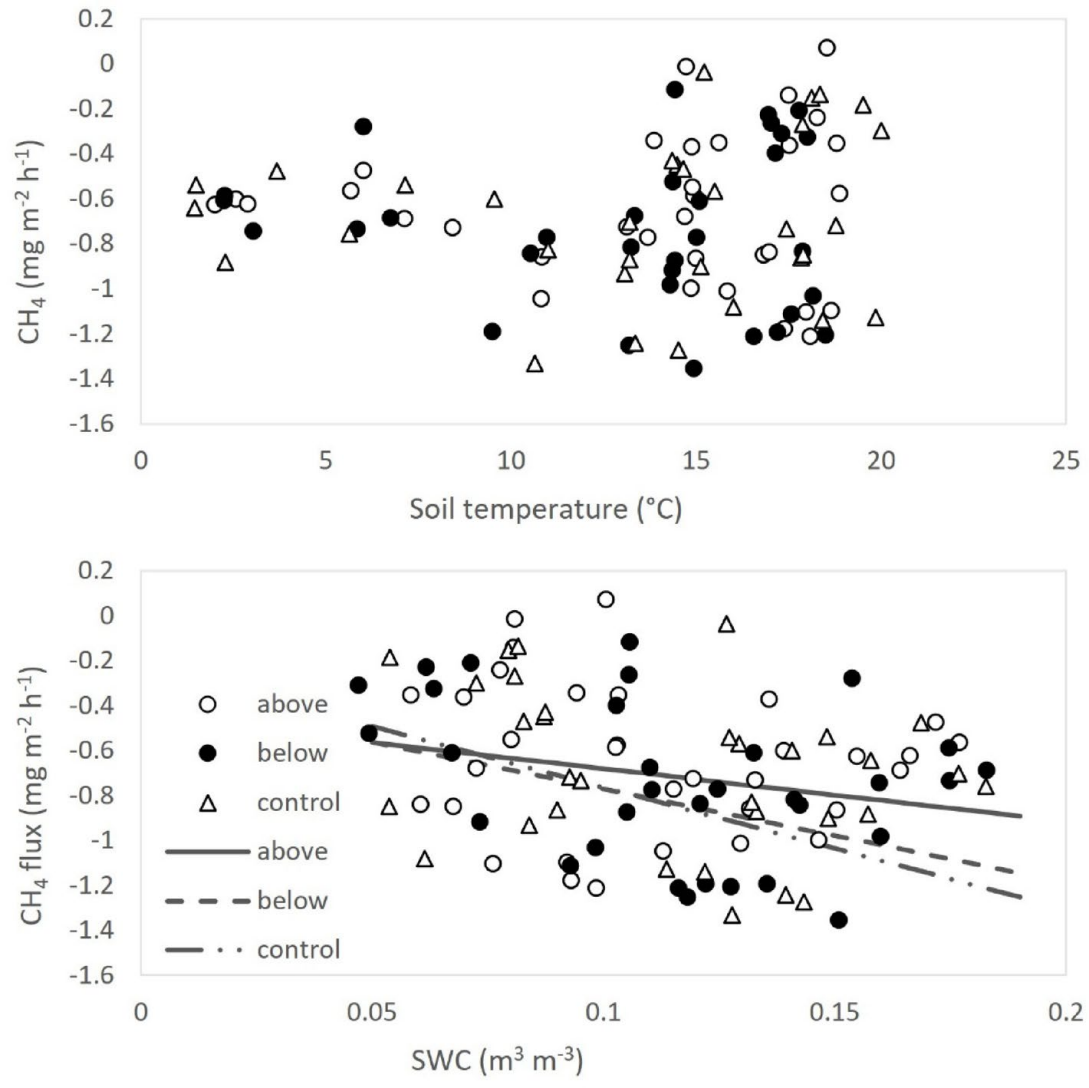
Soil CH_4_ flux in relation to soil temperature (**a**), and soil water content (SWC) (**b**). Positive values indicate net soil GHG fluxes from the soil to the atmosphere, while negative values indicate net fluxes from the atmosphere to the soil. The lines represent the linear model of soil CH_4_ flux in relation to SWC.

A negative soil CH_4_ flux, i.e., an uptake, was detected on all the measurement dates (Fig. 9a), with no clear seasonal variation. No significant relationships were found between soil CH_4_ uptake and soil temperature (Fig. 10a). Soil water content (SWC) showed a weak, albeit significant association with soil CH_4_ uptake, explaining only a small proportion of the variance (R^2^ = 0.04, 0.13, and 0.16 for the above-canopy, below-canopy, and control treatments, respectively; Fig. 10b). Fertilization did not alter the SWC–CH_4_ uptake relationship (Fig. 10b). Cumulative annual CH_4_ flux did not differ significantly among treatments (Fig. 12a).

### Soil N2O emission

**Fig. 11:**
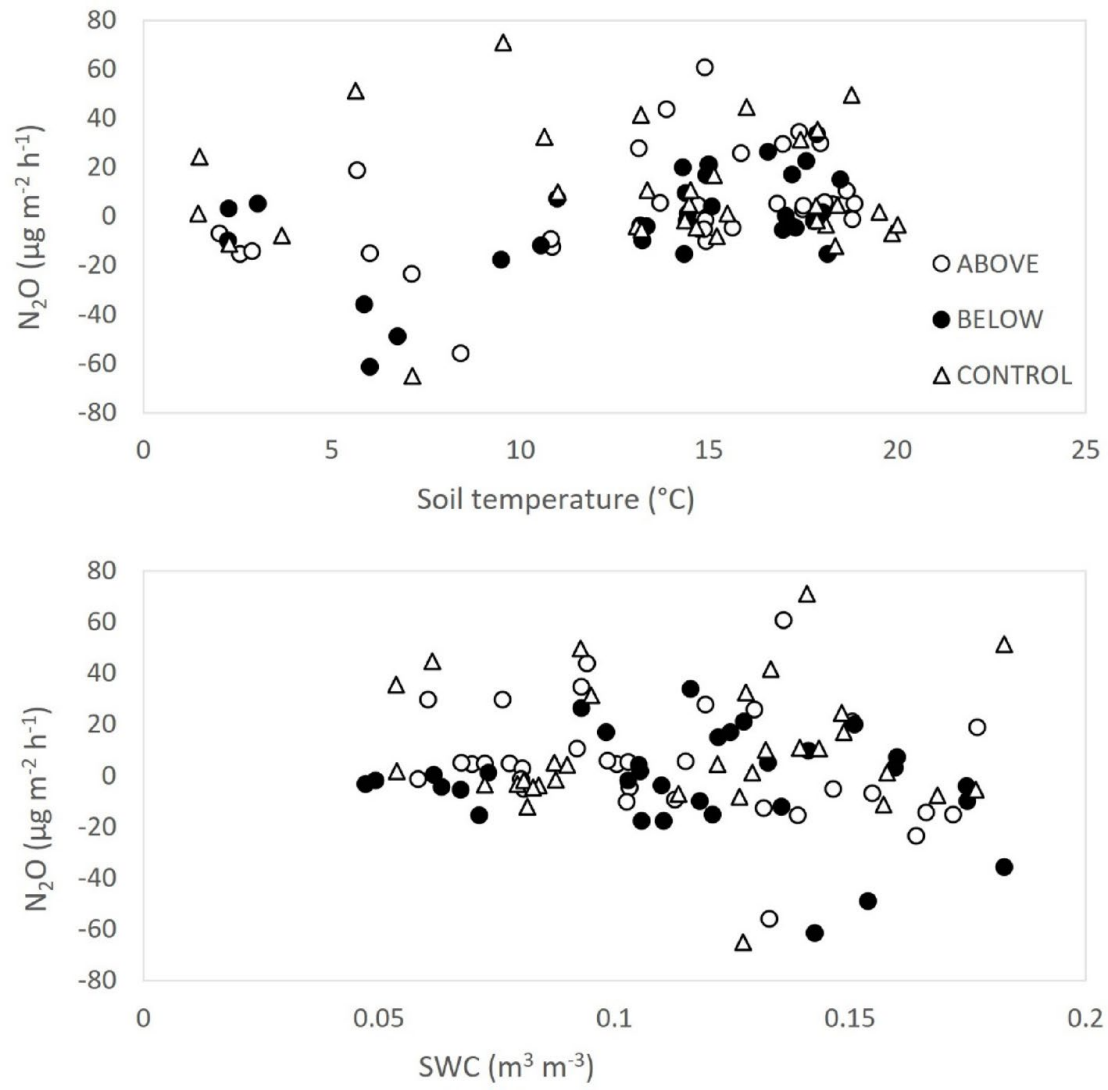
Soil N_2_O emission in relation to soil temperature (**a**), and soil water content (SWC) (**b**).

**Fig. 12:**
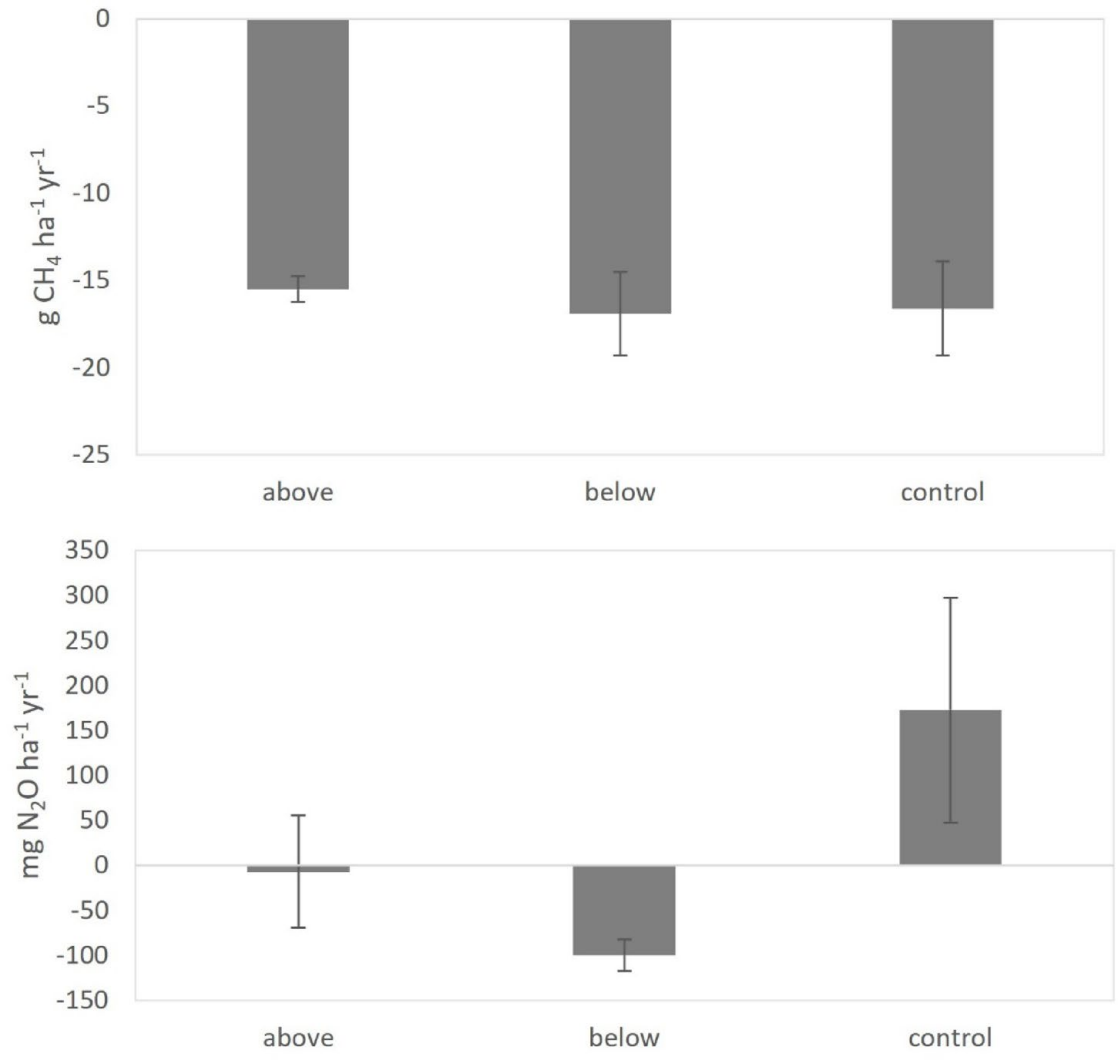
Cumulative annual fluxes of (**a**) CH_4_ and (**b**) N_2_O from the soil. Values are the average of three replicates per treatment; error bars represent the standard error (n = 3).

Soil N_2_O emission ranged from − 48 to 37 μg N_2_O m^−2^ h^−1^. In the last measurement day, a significantly higher N_2_O emission was measured in control in comparison with the treatment below (Fig. 9b). Soil N_2_O emission showed no clear seasonal variation throughout the year. Soil N_2_O emission showed a significant linear relationship with soil temperature (Fig. 11a), but not with SWC (*p* = 0.10, Fig. 11b). The interaction between soil temperature and SWC on soil N_2_O emission was not significant. Annual soil N_2_O emission was higher in control than in treatment above and treatment below (Fig. 12b). However, differences among treatments were not statistically significant.

## Discussion

### Soil N transformation

After five years of fertilization, we observed no lasting change in extractable NO₃^−^ or NH_4_^+^ in either treatment relative to the control, because of a low dosage (close to realistic scenarios for the region) N application. The only clear effect was a rise in topsoil NO_3_^−^ leaching after below‑canopy application towards the end of the study period, in summer 2019 and summer 2020 (Fig. 4). Taken together, these results only partly support hypothesis (i), as additional N increased short‑term N mobility, but not the size of the mineral‑N pool.

Leaching of nitrate could be due to a higher loss of residual N from the first fertilization of the year, which was applied approximately 2 weeks before soil sampling. In fact, a quick increase of N topsoil leaching can be observed soon after the start of the fertilization, although the N saturation is not reached yet^61^. Nitrogen addition may enhance gross nitrification in soil, which in turn increases the accumulation and the leaching of NO_3_-N^62^. However, this mechanism does not explain the higher NO_3_-N topsoil leaching observed in Monticolo, since no positive ΔNO_3_-N was assessed, leading to suppose that the nitrate leaching is a consequence of the quick increase of input explained above. The lack of living roots in the incubated soil cores, and consequently, the absence of N and water uptake by plants, could potentially result in an overestimation of N leaching rates from the topsoil at the experimental site. Nevertheless, our findings suggest that a portion of the N introduced through below-canopy fertilization can readily be lost from the topsoil, possibly elucidating the lack of any discernible effect of fertilization on soil extractable N.

The increase in NO_3_-N topsoil leaching did not occur when N fertilization was applied above the canopy, confirming that the method of N application might, indeed, affect the results of N fertilization experiments. The lack of an increase in soil N leaching in the above-canopy treatment may, therefore, be attributed to the interaction of applied N with the forest canopy^63,64^ and its uptake by canopy foliage^65^. A significant absorption of canopy-applied N was shown also in the same experimental site and in a nearby beech stand, using labelled N fertilization^39,40^. These results suggest that N leaching may have been overestimated in studies in which N was applied directly to the soil. Nevertheless, the delaying effect of the forest canopy on the N reaching the soil, by interception, cannot be ruled out. Yet, NO_3_-N leaching may increase and become more evident over time, with clear differences emerging among the N addition strategies, if the fertilization treatments are repeated for additional consecutive years.

Also, NH_4_-N topsoil leaching was higher when N was applied below the canopy. Both NO_3_-N and NH_4_-N leaching were expected to increase under N addition^28,66^, although at low N input (10–20  kg N ha^−1^ y^−1^) the effect could have been minor^67,68^. However, NH_4_-N leaching in the below-canopy treatment was much lower, due to its lower mobility in soil compared to NO_3_-N^68,69^. In the above-canopy treatment, a portion of NH_4_-N intercepted in the canopy may have also been lost through re-volatilization^6^, further reducing the amount reaching the forest floor and subsequently available for leaching.

In Monticolo, soil N mineralization was reduced after below-canopy N addition but not in the above-canopy treatment. In other studies, N addition was observed to intensify N transformation processes^70^. However, results in which N deposition have an inhibiting effect on N mineralization in forest soil were also reported^71^. Indeed, soil N transformation processes are influenced by N status, dose, and the length of the fertilization period, in addition to soil quality of the experimental site^72^. For example, N addition could reduce soil mineralization by inhibiting microbial activity, especially in temperate forest soils, as in the present case, where N is not a limiting factor for microbial growth^73^. This hypothesis cannot be confirmed in Monticolo, as the soil CO_2_ emissions were not affected by the N application below or above the canopy. However, chronic N application has been shown to decrease the activity of lignin-degrading phenol oxidase in decaying oak leaf litter^74^ or in general high-lignin, low-quality litter^75^. An inhibition in N mineralization is, therefore, consistent with these findings, given that the Monticolo forest is a temperate oak forest, and the presence of canopy may explain the observed variation in mineralization intensity. Other mechanisms have been proposed to explain this inhibitory effect, such as reduced below-ground C allocation due to soil acidification and aluminum release^71^. However, these effects appear in the longterm and probably cannot explain our results. Further studies and longer observation period are needed to determine which of these mechanisms are involved at the Monticolo site, as the few years of available data may be insufficient to determine a structural change in CO_2_ emissions that would support this hypothesis. Anyway, it is important to notice that total N mineralization was not significantly affected in the above-canopy treatment, confirming the importance of including the canopy layer in N manipulation experiments performed in forest ecosystems.

Overall, the extractable NO_3_-N and NH_4_-N observed in the soils of Monticolo were on average slightly lower than the values reported for an oak-dominated mountain forest in China: 7.42 vs. 37.29 and 3.66 vs. 13.95 mg N kg^−1^ soil for NO_3_-N and NH_4_-N, respectively^76^. Extractable NO_3_-N and NH_4_-N contents did not show clear temporal patterns, in both the control and the fertilized plots (Fig. 3). These results are consistent with laboratory experiments in which a one-time N addition of 40 kg N ha^−1^ yr^−1^ to soils from two forest sites did not increase extractable NH_4_^+^-N or NO_3_^−^-N in one of the sites^68^. By contrast, a 5-year study in a temperate forest, where higher N inputs (50 and 150 kg N ha^−1^ yr^−1^, i.e., 3–8 times those in the present study) were applied, reported clear accumulation of both extractable NO₃-N and NH_4_-N in the soil^77^. A meta-analysis highlighted a significant increase in soil mineral N content after N addition, with a larger influence on NO_3_-N than on NH_4_-N^24^. In the soils of a Korean pine plantation, N additions (20, 40 and 80 kg N ha^−1^ yr^−1^) increased extractable NO_3_^−^-N but not extractable NH_4_^+^-N, with the accumulation of soil NO_3_^−^-N attributed to internal soil N transformations^11^. A lack of differences between N fertilization treatments, which bring us to partially reject our first hypothesis, can be explained by the loss of the added N by N leaching from topsoil or microbial N immobilization in the below-canopy treatment^78^, or potentially by canopy revolatilization and leaf direct uptake in the above canopy treatment^6,39^.

### Soil GHG fluxes

Soil GHG fluxes showed high temporal variability regardless of the N loads, in agreement with previous similar experiments performed in forest ecosystems^36,56^. No effect of treatment was evidenced, for all measured gases, leading us to reject the second hypothesis. Specifically, in the present study, soil CO_2_ flux was not influenced by any of the N addition methods, with values ranging from 0.11 to 0.42 g CO_2_ m^−2^ h^−1^ throughout the sampling period (Fig. 6). These values are lower than the average soil CO_2_ flux reported for temperate forests^79^. The average annual cumulative soil CO_2_ flux was 3806 and 3863 g CO_2_ m^−2^ y^−1^ in the first and the second year, respectively. These values are slightly higher than those reported by Giasson et al.^80^ for five different vegetation types in a temperate forest ecosystem (1719–3487 g CO_2_ m^−2^ y^−1^), while the annual soil CO_2_ flux in a mixed-deciduous forest reported by Bowden et al.^81^ was more than double (1097 and 1366 g CO_2_ m^−2^ y^−1^ in 2 years, in control conditions).

In Monticolo, the absence of significant effects of N fertilization (either above or below the canopy) on soil CO_2_ flux could be due to the relatively low N loads (not sufficient to augment the available N) and to the high variability in CO_2_ fluxes. The influence of N loads on soil CO_2_ flux may follow a threshold-type response to N accumulation in forest soils^82^; therefore, significant effects may only become evident over the long term, following repeated N applications.

The literature reports contrasting effects of nitrogen (N) application on soil CO_2_ fluxes, likely due to differences in ecosystem type, experimental duration, and fertilization regime. For instance, in boreal coniferous forests it has been found that long-term N fertilization significantly reduced soil CO_2_ emissions across all treatment levels compared to controls, while simultaneously enhancing carbon sequestration in tree biomass^36^. These findings highlight the potential for N addition to mitigate net greenhouse gas emissions, despite a moderate increase in soil N_2_O emissions. In the present study, lack of a significant effect of N application on soil CO_2_ flux is in line with a meta-analysis^24^, which determined that soil CO_2_ flux decreases significantly under N addition in boreal and tropical forests, without changing significantly in temperate forests. Other studies, however, reported the opposite. Another meta-analysis concluded that soil CO_2_ flux decreases under N addition, although no distinction among biomes was made^25^. Likewise, it has been indicated that N deposition significantly reduces soil CO_2_ flux in boreal and temperate forests, but not in tropical forests^83^. Another possible reason lies in the fact that soil CO_2_ flux results from both heterotrophic (microbial) and autotrophic (root) respiration, which may respond differently to N addition. In the Monticolo oak stand, characterized by lignin-rich litter, heterotrophic respiration is expected to decrease particularly under below-canopy N addition, due to reduced microbial biomass and activity^10,81,84^. Autotrophic respiration, however, may increase^85^ or decrease^73^, as its drivers remain unclear. While some studies link it to increased fine root biomass under N addition^84,85^, others report the opposite trend^86^. Our method did not allow for distinguishing these components, preventing us from determining whether the lack of a visible N effect on soil CO_2_ flux resulted from opposing responses of heterotrophic and autotrophic respiration. Soil respiration is affected by different environmental factors, among which the most important are soil temperature and soil moisture^60,87–89^. In Monticolo, soil CO_2_ flux was limited by low soil moisture during summer and by low soil temperature during winter. The effect of soil temperature and soil moisture may influence soil respiration more than N addition, therefore, overcoming the effect of N^90^. However, no effect of N was detected even when soil temperature and soil moisture were considered in the model. In fact, the relationship between soil CO_2_ flux, soil temperature, and SWC was not affected by fertilization treatment. Furthermore, the sensitivity of soil CO_2_ flux to soil temperature was not affected either, even in non-limiting soil moisture conditions.

In general, soil CH_4_ flux measured in Monticolo was negative, indicating a net uptake of CH_4_ by the soil. Soils in aerobic environments are characterized by methanotrophic conditions, determining the uptake of CH_4_ by microbial communities^91,92^. Soil CH_4_ uptake was not significantly affected by treatments. Higher NH_4_-N availability may inhibit the reduction of CH_4_^93^, while NO_3_-N at high concentration interferes with the synthesis of enzymes involved in CH_4_ oxidation^94^. Nevertheless, in several studies, soil CH_4_ uptake was found to both increase at low N load and decrease at high N supply^94,95^. Indeed, NO_3_-N at low concentration may promote microbial activity and consequently soil CH_4_ uptake^94^. A non-linear response of soil-atmosphere CH_4_ fluxes to N application has been suggested in a field experiment in Mediterranean conditions, with intermediate levels (10 kg N ha^−1^ y^−1^) of N fertilization having the most negative impact on ecosystems to act as sinks of atmospheric CH_4_^56^. The positive relationship observed between soil CH_4_ uptake and SWC found in Monticolo agrees with results by Serrano-Silva et al.^92^, who observed that, at SWC < 20%, CH_4_ consumption rates decreases, as also the methanotrophs are inhibited at this SWC. On the other hand, the lack of correlation between soil CH_4_ uptake and soil temperature in our site can be due to prevailing soil moisture over soil temperature effects, despite soil temperature was previously reported as a significant driver of soil CH_4_ uptake^92,93^.

Soil N_2_O emission measured in Monticolo was very low and did not show any significant difference among treatments, except on one date, when the N_2_O emission was higher in control and lower in the fertilized plots. Other studies reported increases in soil N_2_O emission because of N deposition or N fertilization^36,96^, detectable also at low N loads (< 50 kg N ha^−1^ y^−1^) and short-term scale (< 5 years)^97^. An increase in soil N_2_O emission was also detected in laboratory experiments, but not in field conditions (both conifer and broadleaved forests)^98^. Yet, an increase in soil N_2_O emission was determined at a short timescale, followed by a decrease at a long timescale or with a high N deposition rate^71,96^.

Furthermore, no significant effects from other environmental drivers were detected in the present study, as soil N_2_O emissions showed no response to soil temperature or soil moisture—factors considered the primary environmental drivers of soil N_2_O emissions^71^. The high variability in these fluxes, ranging from negative to positive values, may have masked potential treatment effects, explaining the absence of a clear N response. We cannot exclude that the absence of significant correlation with SWC is related to the fact that the SWC was measured in the center of the plot and not in the collars used for N_2_O fluxes. Even if plots were chosen in relatively homogeneous areas in the forest, to ensure good representativity of the plot conditions, the microvariability of soil conditions between measuring points may have introduced a further disturbance effect. However, the absence of relationship with SWC could also be due to the shallow soil and the low clay content, likely favoring fast water drainage, where anaerobic conditions favorable to denitrification and N_2_O emissions only rarely occur. Therefore, only continuous measurements of N_2_O fluxes could have evidenced differences in the emission of this gas during or immediately after rainfall events.

Few studies compared the effects of N input on soil GHG fluxes in above- and below-canopy fertilization treatments. An increase in soil N_2_O emission when N was applied below-canopy at a rate of 50 kg N ha^−1^ y^−1^, but not at a lower application rate (25 kg N ha^−1^ y^−1^)^13^. This result was related to the increase of soil N concentration in below-canopy fertilized plots, which did not occur in Monticolo.

Considering the high heterogeneity which usually characterizes forest soils, we acknowledge that measuring GHG fluxes on 3 collars for plot and three plot per treatment, i.e. 9 collars for each treatment, may not have been sufficient to detect the effect of treatments on GHG fluxes. The number of measurement points chosen for each plot represents a trade-off between the need to account for the high spatial variability of forest soil and the need to perform the measurements in a relatively short period. Adding more measurement points to each plot would better capture spatial heterogeneity in root distribution and soil conditions within the plots but would also introduce heterogeneity in GHG measurements of the forest, due to the diel variation in soil temperature and plant metabolic activity which affect measured soil GHG fluxes^29^.

The absence of a significant effect of N fertilization on soil N_2_O emission observed in the Monticolo forest is coherent with the lack of treatment effects on extractable soil N. In fact, N pools in Monticolo did not differ significantly between treatments^39^. However, given the lower N mineralization observed in the below-canopy treatment, an increase of N_2_O emissions could be expected in the same treatment. In fact, N_2_O is a product of both nitrification and denitrification processes occurring in soils^99^ and long-term N addition may worsen conditions that favor the denitrification activity, by reducing soil pH, decreasing C availability, and enhancing NH_4_-N concentration^71,96,100^. The effect on N mineralization in Monticolo may not have been strong enough to produce a measurable impact on N_2_O emissions, given the low fluxes observed even in the control plots. Significant effects might, however, be detectable over the long term if N applications are repeated, and N saturation is eventually reached.

## Conclusion

Our study reveals that in a sessile oak forest, the N fertilization at a rate of 20 kg N ha^−1^ y^−1^ had contrasting effects depending on the application method. In fact, topsoil N leaching increased, and N mineralization decreased under below-canopy fertilization, but not when fertilization was applied above the canopy. This suggests that the interaction (interception and/or absorption) of added N with the canopy could delay or change its effects on forest ecosystems. This finding aligns with previous studies and highlights the importance of including canopy in N manipulation experiments.

The increased N input did not influence the fluxes of CO_2_, CH_4_ or N_2_O from soil, either applied above or below the canopy, even if the potential effects of environmental variables (soil temperature and moisture) were considered. While in the below-canopy application this may be due to the loss of added N through soil leaching, in the treatment above, the interception and absorption of N by the tree canopy may have reduced the amount of N reaching the soil, as evidence by previous studies in the same area, and therefore its effect on soil microbes. Further studies are needed to assess the mechanisms involved at the site examined. Furthermore, given the relatively short experimental period (6 years), and the relatively low N dose applied, the continuation over the long term of N manipulation experiments including above-canopy applications is foreseen, to assess if canopy-N interactions could delay or reduce N saturation in forest ecosystems.

## Data Availability

The data supporting the findings of this study are available from the corresponding author, upon reasonable request.
